# Internal adaptation assessment of implant infrastructures manufactured through five different techniques (heat-press, milling, lost wax, calcinable cylinder, and CAD/Waxx®): an *in vitro* pilot study

**DOI:** 10.3389/fdmed.2024.1483177

**Published:** 2024-11-06

**Authors:** Régis Sartori, Juliana Campos Hasse Fernandes, Gustavo Vicentis Oliveira Fernandes, Julio Cesar Joly

**Affiliations:** ^1^ Department of Implantology and Periodontology, Faculdade São Leopoldo Mandic, Campinas, Brazil; ^2^ Private Practitioner, St. Louis, MO, United States; ^3^ Missouri School of Dentistry & Oral Health, A. T. Still University, St. Louis, MO, United States

**Keywords:** dental marginal adaptation, dental prosthesis, dental porcelain, CAD/CAM, prosthetic abutment

## Abstract

**Introduction:**

The aim of this study was to evaluate the fit performance of implant infrastructures manufactured by five different techniques: heat-press (IPS), milling (ZIR), lost wax (CER), calcinable cylinder (CAL), and CAD/Waxx® (CAD).

**Methods:**

The methodology was based on the Replica Technique, which can simulate and evaluate the fit of the infrastructure on the implant component. Thus, each infrastructure was internally filled with low-viscosity silicone addition and seated on the component until its final setting, obtaining the replica of the cementation space. After removing the coping containing the silicone film, light-density silicone was inserted addition in place of the components, and in its surroundings, condensation silicone was applied, establishing support for the assembly. The joint was sectioned mesiodistally, photographed, and analyzed in image processing software in order to measure the thickness of the interface infrastructure/implant at five different areas: marginal opening (M), gingival-axial angle (G-A), axial region (A), axial-occlusal angle (A-O) and occlusal surface (O).

**Results:**

The lowest and the highest average thickness between groups was, respectively, IPS: 187.5 μm and CAD: 221.6 μm, with statistically significant differences (*p* < 0.01) among all five groups; the lowest and the highest average of all groups in each point was, respectively, A: 99.86 μm and O: 279.78 μm. The IPS group exhibited the lowest value of the internal space of the infrastructure on the implant. The marginal region of all groups demonstrated a correlation with the findings in the literature, except the CAL group; otherwise, the occlusal region and the angles A-O and G-A exhibited values beyond that expected.

**Discussion:**

It was possible to conclude that the five infrastructure groups presented different adaptations, suggesting possible interference in the internal spaces due to the manufacturing infrastructure processes.

## 1 Introduction

The esthetic and functional search for compromised teeth capable of recovery has driven Restorative Dentistry (1). Esthetic and functional dental rehabilitations have adopted ceramics as a favorite material for crowns and prosthetic infrastructures. This choice is based on its ability to mimic dentin tissue and tooth enamel, concomitantly with the resistance and biocompatibility offered (2, 3). Otherwise, ceramics are materials prone to cracking due to the possible presence of microscopic defects, and their resistance to fracture is limited and time-dependent since the aqueous environment of the oral cavity causes the material to corrode. Furthermore, the conditions of dental preparation and the cementing technique of the pieces also affect the resistance of the ceramic crown (4). Then, while developing several alternative materials, incorporating zirconia in dental ceramics has improved mechanical properties without significantly altering the material's esthetics (5, 6). At the same time, new systems for manufacturing ceramic prosthetic parts have emerged (7–9).

Prosthetic parts made with dental ceramics can be obtained through different processes, such as pressing or injection, milling, and/or sintering. The choice of manufacturing method depends on how the ceramic is presented, whether in powder, tablet, or block form (9). In the pressing or injection process, ceramic tablets are used, which are injected into a coating through high temperatures and pressure, as occurs in the lost-wax technique. In this case, ceramic tiles reinforced with lithium disilicate can be used to provide greater resistance to the final piece; however, they present esthetic losses due to the accentuated opacity (9, 10). Given technological advances, the computer-aided design and computer-aided manufacturing (CAD/CAM) system was developed to provide quality, speed, and greater accuracy in producing prosthetic parts, ensuring the most precise adaptation compared to conventional technologies. This system allowed the manufacture of ceramic crowns through two different processes: the single-layer (or monolithic) type and the double-layer type (11, 12).

Therefore, the submitted heating process causes some microscopic distortion levels in the material, resulting in the maladaptation or internal spaces of the prosthetic part (12). The longevity and clinical success of ceramic restorations depend on adequate marginal and internal adaptation of the crowns to the preparations (13–15). Gonzalo et al. (16) evaluated two groups of ceramic infrastructures manufactured by two different CAD/CAM systems (Procera® Bridge Zirconia and Lava® AllCeramic System); both groups presented clinically acceptable values, ranging from 26 ± 19 µm (Procera® System) to 76 ± 36 µm (Lava® System). In the study carried out by Tinschert et al. (17), the marginal adaptation values ​​found were 60.5 µm to 74.0 µm; marginal openings ranged from 42.9 µm to 46.3 µm; vertical discrepancies from 20.9 µm to 48.0 µm; and horizontal discrepancies from 42.0 µm to 58.8 µm. These findings were obtained by *in vitro* evaluations of fixed partial dentures manufactured by the Precident DCS system. Korkut et al. (18) compared marginal and internal adaptations of infrastructures manufactured by the Cercon® and IPS Empress II systems; the authors found lower adaptation values ​​in the Cercon® group (marginal: 43.2 µm; internal: 57.1 µm) and higher in the IPS Empress II group (marginal: 47.5 µm; internal: 74.01 µm), being all clinically acceptable values. Guzelian (19) considered that marginal and internal openings of up to 120 µm are acceptable in clinical practice, as observed in Beuer et al.'s (2009) and Yeo et al.'s (2003) studies.

Other authors extrapolate clinically acceptable values, considering the range from 86 µm to 154 µm adequate (20) and internal variation of 41 µm to 141 µm as satisfactory (21). Jacobs and Windeler (22) investigated the relationship between the dissolution rate of zinc phosphate cement and the degree of marginal opening of prosthetic parts measuring 25 µm, 50 µm, 75 µm, and 150 µm; they concluded that the dissolution of cement did not depend on the degree of exposure when the opening is smaller than 75 µm. However, as the marginal opening increases, cement degradation also increases. The value of clinically acceptable marginal adaptation should vary between 100 µm and 150 µm; the marginal thickness value accepted for the CAD/CAM system ranges between 50 µm and 100 µm. However, the internal adaptation must be less than 70 μm to maintain adequate resistance to fractures of ceramic crowns (13, 23).

Several techniques are available for evaluating the marginal and internal adaptations of the crown/preparation interface, such as direct vision, exploratory visual examination, micrometric measurements, profile projector, scanning microscopy (conventional, electronic, or digital), laser videography, and printing techniques (13, 23, 24). The replica technique consists of reproducing the cementation of prosthetic parts for subsequent analysis of the marginal and internal adaptation without the need to destroy the crowns, commonly used to test restorations’ internal fit; it offers a non-destructive, simple, less expensive, easy, and reliable method with acceptable accuracy, which can be used *in vitro* and *in vivo* (13, 25–28).

The cementation simulation is based on depositing a layer of elastomeric impression material. Generally, addition silicone is used on the internal surface of the infrastructure or prosthetic crown, being positioned on a specific preparation. A predefined static load device is placed on this system in order to standardize the settling time and pressure of the parts (12, 13, 29–31). Another addition silicone, of a different color, is applied inside the infrastructure – containing the first film – to stabilize the replica, thus simulating the conformation of the prosthetic abutment. Both silicones are removed from each infrastructure, incorporated into epoxy cylinders, and filled with condensation silicone of a different color. The samples are cut axially in the mesio-distal and bucco-lingual direction, aided by cutting guides previously made from epoxy material (32, 33).

In Laurent et al.'s study (2008), the authors validated using silicone to replicate the space between the tooth and the inner surface of prosthetic crowns. They concluded that using silicone makes it possible to measure the cementation film between the prosthesis and tooth preparation, regardless of the location analyzed (cervical, occlusal, or axial). Generally, discrepancies between the preparation and infrastructure in axial and cervical sites are evaluated using microscopy or another of the abovementioned techniques. Measurements can also be carried out in the cusp tip regions and central occlusal regions (33).

Thus, due to the many types of materials used to develop a prosthetic framework and the need to control internal adaptation, which can easily vary for each type of material, the objectives of the present pilot study were to measure and compare the internal adaptation of five types of infrastructures through the Replica Technique. The null hypothesis was that no statistically significant difference was observed among the groups.

## 2 Materials and methods

The experimental design was based on Lee et al.'s (2008), Paes's (23), and Colpani et al.'s (2012) studies, using ceramic and metallic infrastructures made on prosthetic abutment as *in vitro* research. The pieces were subjected to evaluation of the cementation film through the replica technique, and the procedures were conducted in a laboratory environment at Sao Leopold Mandic – Faculty of Dentistry (Campinas, SP, Brazil) between 08/2020 and 06/2021. The groups were: two metal-free: (1) one in zirconia using the CAD/CAM system (Cercon/Dentsplay®) and (2) another in lithium disilicate using the injected technique (e-max/Ivoclar/Vivadent®); and three cast metal infrastructures: (3) one using the Conventional Technique, (4) CAD/Waxx® system (Vita CAD-Waxx® for InLab) associated with the Sirona® CAD/CAM system, and (5) prefabricated and subsequently cast castable cylinders.

### 2.1 Preparation of the set

**Figure 1:**
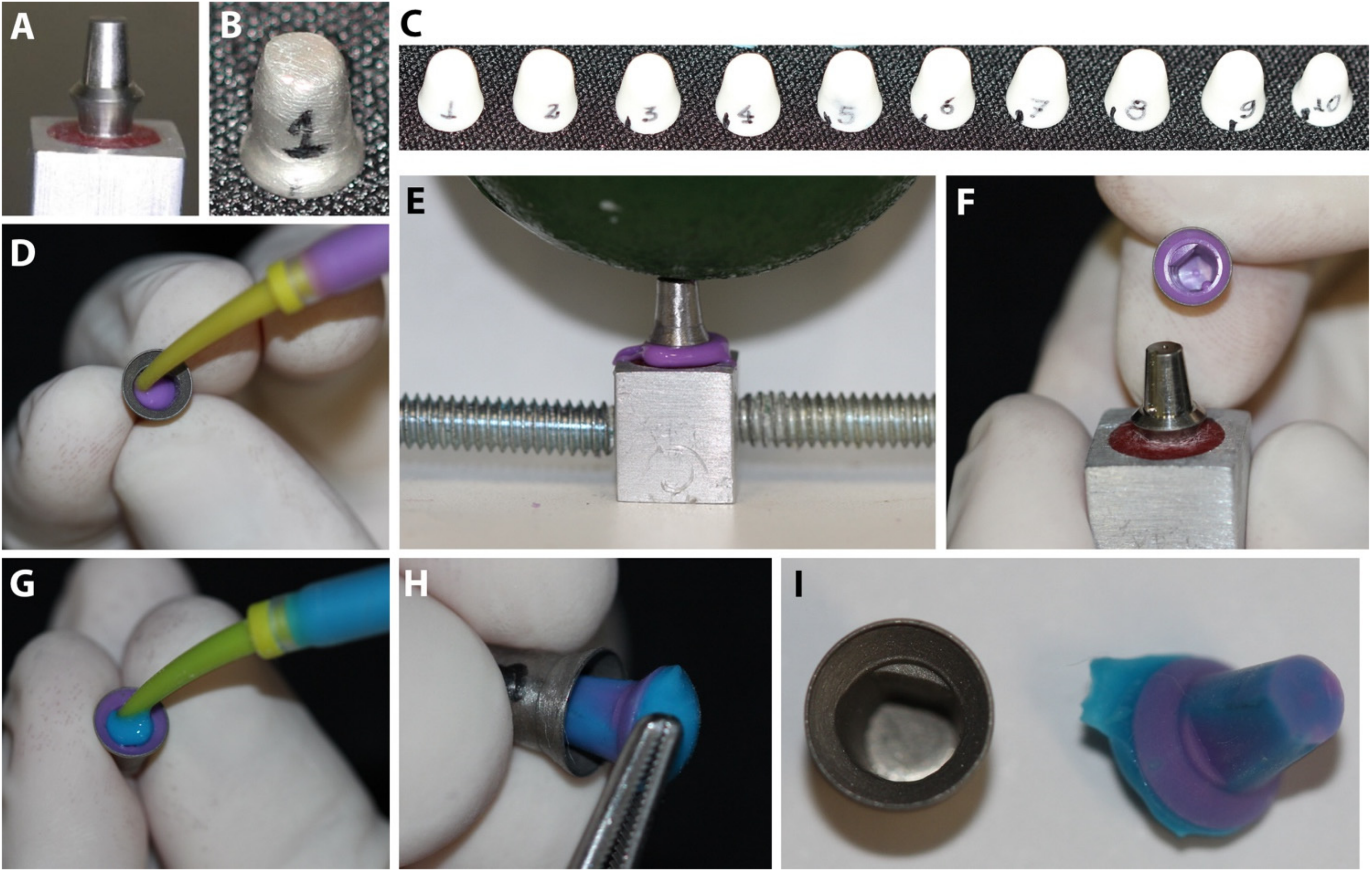
**(A)** Set of the implant and abutment; **(B)** metallic infrastructure; **(C)** zirconia-reinforced ceramic (ZIR) infrastructures; **(D)** Low viscosity addition silicone injected into the internal portion of the infrastructure; **(E)** infrastructure placed on the component under standardized compression (static load of 20 N.cm for five minutes); **(F)** framework containing the silicone film removed from the surface of the prosthetic abutment; **(G)** second application of medium-viscosity silicone creating a firm internal support body; **(H and I)** the assembly was delicately removed.

An aluminum base was made with the following dimensions: 112 mm × 15 mm × 13 mm, with a circular opening measuring 10 mm in diameter and 12 mm in depth in the center of the upper part, where the 6.5 mm × 12 mm titanium platform implant from the Globtek® brand (Morse Taper, Globtek Implant System®, Korea) was fixed. A milling machine (1000N BioArt® Milling Machine, BioArt® Dental Equipment São Carlos-SP, Brazil) was used for this fixation in order to leave the implant completely perpendicular to the horizontal plane. Chemically activated acrylic resin (Trim Plus Red-Pattern Acrylic, Boswarth Company®, USA) was then added to the inside of the aluminum base to help stabilize the implant. After polymerization of the acrylic resin, a solid abutment of 4.3 mm × 5.5 mm from the same manufacturer was installed on the implant, with a torque of 30 N. For this, a torque wrench from the manufacturer was used to prepare the implant and abutment set (Figure 1A) (34).

Using a second aluminum base and the same method described, another die was made with the analog of the abutment from the same manufacturer. It was made of titanium and has dimensions of 4.3 mm × 5.5 mm, with a cervical termination in an inclined step of 135°. The purpose of making these two test bases is justified because the frameworks are made in the laboratory on an analog and cemented in the mouth on the abutment itself.

### 2.2 Making the frameworks

Fifty frameworks (*n* = 50) were made and divided into groups following: (A) 10 frameworks in ceramic reinforced with lithium disilicate (IPS, e-max, Ivoclair Vivadent®); (B)10 frameworks in ceramic reinforced with zirconia (CAD/CAM, Cercon, Dentsply®); (C) 10 cast metal frameworks, obtained from wax patterns using the immersion technique, followed by the conventional lost-wax technique; (D) 10 cast metal frameworks, obtained from milled acrylic patterns (Vita CAD, Waxx® for InLab) associated with the Sirona® system (CAD/CAM), followed by the conventional lost-wax technique; (E) 10 metal frameworks cast from prefabricated castable cylinders, followed by the conventional lost wax technique.

#### 2.2.1 Fabrication of lithium disilicate-reinforced ceramic frameworks (IPS)

Ten 0.5 mm thick caps were constructed on the analog abutment using the Nealon technique in chemically activated liquid acrylic resin (Pattern Resin LS® acrylic resin, GC Restorative Dentistry, U.S.A.). The cervical region of each cap was covered with cast wax (Al Dent Wax, Al Dent, DentalProdukte®, Germany) using a dropper. The caps were removed from the analog abutment and separated to continue the laboratory inclusion stage.

The inclusion process was carried out by fixing a wax sprue (Wax Sprue No. 3, Kota Imports®, Brazil) on each cap, joining them to the inclusion ring (Inclusion Ring, Ivoclar/Vivadent AG®, Liechtenstein) and another sprue on the opposite side of the rim, with the same measurement, for compensation. Both parts of the inclusion ring were isolated with petroleum jelly (Solid Vaseline, LBS Laborasa®, Brazil) using a brush (Tigre brush n. 2, Pincéis Tigre® S/A, Brazil). The investment material (Gilvest HS powder/liquid, BK Giulini®, Germany) was spatulated (Vacuum Spatula, Polidental® Ltda. Cotia-SP, Brazil) under vibration (Vibro 3 Rivoli Vibrator, Rivoli® Ind. e Com. Ltda., Brazil) for 1 min; was introduced into the inclusion ring, filling it up to the height of the sprues.

After setting, the ring was removed, and the investment cylinder was placed in an oven (Vulcan 3-130 Dentsplay® oven, Degussa Dental, Germany), preheated to 850°C, and kept there for 1 h. The cylinder was then removed from the oven and cooled for 25 min. The next step was injecting the ceramic to replace the wax and acrylic. The ceramic tablet reinforced with lithium disilicate (e-max Press® tablet, Ivoclar/Vivadent AG®, Liechtenstein) and the plunger were positioned in the upper part of the cylinder. The assembly was placed in the oven (EP 600 oven, Ivoclar/Vivadent®, Liechtenstein), with a cycle and temperature set at 18 min, at 915°C, promoting the injection of the tablet into the cylinder.

In the disinclusion stage, the lining was sawed (Starrett hand saw, Starrett® Ind. e Com. Ltda., Brazil) and, on its surface, aluminum oxide was blasted (Aluminum oxide 320, DentFort Crismar® Com. Ltda., Brazil), until the infrastructure was obtained. The thickness of the infrastructures was checked using a thickness gauge, and the excess was removed with diamond discs.

#### 2.2.2 Production of metal infrastructures using the Conventional Lost-Wax Technique (CER)

From the analog pillars, wax patterns were constructed using the Immersion Technique and subsequently cast using the Conventional Lost-Wax Technique. To do it, the analog was immersed in cervical memory wax (INOWAX, Formaden®, Brazil), and then another wax (AESTHETIC, Formaden®, Brazil) was used to make the necessary corrections. To make the cast, each pattern was first carefully removed from the die and joined to a feeding channel (SprueWax, Formaden®, Brazil), aiming to provide a path for the molten metal to reach the space left by the pattern. This assembly was supported by a silicone-forming base and surrounded by a ring, also made of silicone.

The inclusion process was then started. For this, a correct water/coating powder ratio was required. The powder was poured over the water and spatulated (Easy Mix vacuum spatulator, BEGO®, Germany) until all the powder was wet. Then the coating (Bellavest SH, BEGO®, Germany) was slowly poured into the ring to fill it completely, and, after its final setting, the wax pattern and the feeding channel were eliminated by means of a high-temperature furnace (KNEBEL®, Brazil), a process called burning the pattern. In this way, the resulting space was filled by the molten alloy (Wironia Light, BEGO®, Germany), forced by pressure through the feeding channel utilizing a centrifuge (EDG Induction, Brazil), thus obtaining the cast (35).

#### 2.2.3 Manufacturing of metal frameworks using the Vita CAD-Waxx® system (CAD)

The use of this system requires a burnable acrylate polymer block that leaves no residue. To obtain the parts, it was first necessary to scan the analog using the In Lab 3D® software (Sirona, Dental System®, Germany). This technology allows for determining the desired space between the infrastructure/prosthetic abutment interface. In the present study, it was decided to test three different internal spaces: 60 µm, 70 µm, and 90 µm. The 60 µm space did not allow the infrastructure to fit over the abutment; it means that the internal space was too small, preventing its placement; the 90 µm space presented a large misfit; and when applying 70 µm, an adequate fit was observed, and this was the thickness selected to manufacture the 10 infrastructures using the CAD/Waxx® system. Sequentially, a block of incinerable acrylate polymer (VITTA®, Germany) was placed in a milling machine (Sirona®, Dental System, Germany) to then begin the milling process of this block.

After milling the piece, the excess was removed using a tungsten carbide cross-cut drill (Edenta®, Switzerland), leaving the acrylic patterns ready for casting. The casting stage of the incinerable acrylate patterns used the same methodology described previously for casting the wax patterns.

#### 2.2.4 Manufacturing of metal frameworks from castable cylinders (CAL)

Ten castable frameworks (Plastic Coping, DIO Implant®, Korea) were commercially obtained and sent to the prosthesis laboratory to be cast in a chrome-cobalt (Cr-Co) metal alloy (Figure 1B). The casting steps followed the conventional lost-wax technique.

#### 2.2.5 Manufacturing of zirconia-reinforced ceramic (ZIR) infrastructures

The same analogous abutment mentioned above was used to manufacture the zirconia-reinforced ceramic infrastructures, and the first step consisted of scanning it. To this end, reliefs were made of the sharp angles of the abutment with wax (Schuler Wax, Schuler Dental®, Germany), according to the manufacturer's instructions, since the scanner is not capable of scanning these areas. Silver powder (Scan Powder, Degussa® Dental, Germany) was also added to prevent the reflection of the laser from the scanner (Cercon Eye Dentsplay® Reader, Degussa® Dental, Germany). A plaster base containing wax in the center was used to fix the die, which was then centered on the Cercon® Eye scanner support to start scanning.

The adjustments for the end, internal relief, and thickness of the frameworks were duly determined by the computerized digital system (Degudent, Cercon® System, Germany). Since the sintering step results in a dimensional reduction of the parts of around 30%, the system itself compensates for this contraction and mills the framework 30% larger. Then, the zirconia disc (Cercon Dentsplay® Zirconia Disc, Degussa® Dental, Germany) was positioned in the milling machine (Cercon Brain Expert Dentsplay® Milling Machine, Degussa® Dental, Germany), accompanied by its code on the USB stick. The milling of the framework began with the constant automatic exchange of milling cutters, the last of which was a low-speed laminated type, in order to detach the disc. A pink rubber (Cerapol Pink Diamond Finishing Rubber, Edenta®, USA) was used to finish the parts.

The subsequent step was the sintering process of the frameworks. The pieces were placed in a special container containing alumina spheres, then taken to the oven (Cercon Heat Plus Dentsplay® Sintering Oven, Degussa Dental, Germany) and kept for approximately eight hours and fifteen minutes at a temperature of 1350°C. A new finish was performed with a drill (Diamond tip n. 92) and diamond rubber (Eurodental Blue finishing diamond rubber) at low speed to finish the framework (Figure 1C).

### 2.3 Replica technique

The frameworks were cleaned with cotton (Soft Plus, Orlando Antonio Bussioli® ME., Brazil) and a 70% alcohol solution (Álcool Mega 70° INPM Hospitalar, Mega Química® Ind. e Com. Eireli, Brazil). The samples from each group were numbered from one to ten on the external face, wrapped in cotton, and placed in small paper boxes.

Low viscosity addition silicone (Express XT Light Body, 3M ESPE®, St Paul, MN, U.S.A.) was injected into the internal portion of each part using mixing tips attached to the dispensing gun (Universal Dispensing Gun, Nova DFL®, Brazil) (Figure 1D). Each framework containing the impression material was placed on the component under compression and a static load of 20 N.cm was applied to the assembly for five minutes in order to standardize the setting time and pressure during the silicone setting (Figure 1E). The excess silicone deposited beyond the margins was removed with a scalpel blade (Solidor Scalpel Blade, Suzhou Kyuan Medical® App. Co. Ltd., China) and discarded. The framework containing the silicone film was carefully removed from the surface of the prosthetic abutment (Figure 1F).

Medium viscosity addition silicone (Express XT Regular Body, 3M ESPE®, St Paul, MN, USA) was added to its interior using the gun and mixing tips mentioned above. The second application of medium-viscosity silicone was necessary to create a firm internal support body for the low-viscosity silicone film (Figure 1G). This assembly was delicately removed from inside the framework to receive another support base formed on the external surface of the silicone film (Figures 1H,I).

**Figure 2:**
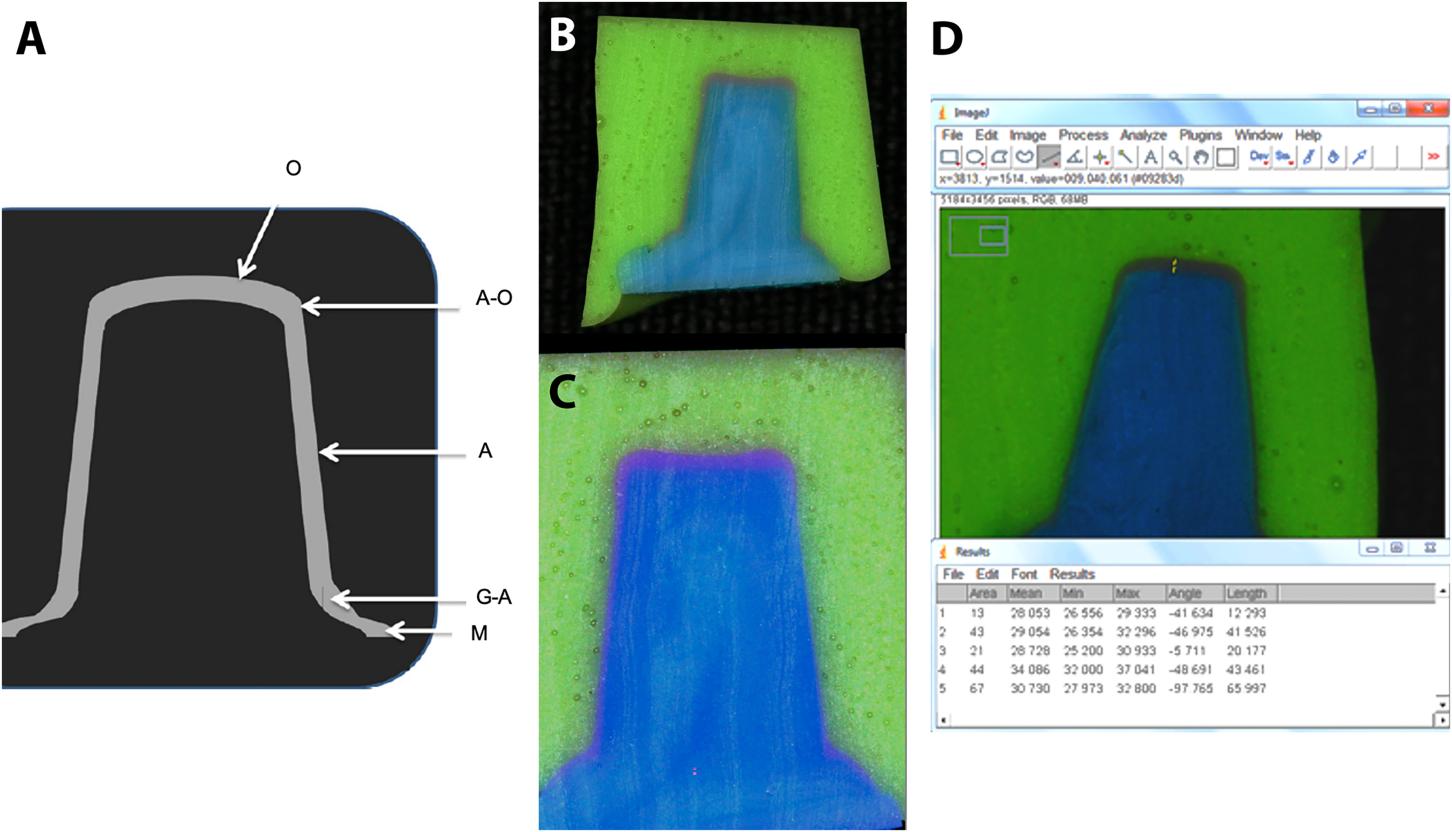
**(A)** Points analyzed: marginal opening (M), gingival-axial angle (G-A), axial region (A), axial-occlusal angle (A-O), and occlusal region (O); **(B–D)** adjustment of the set to be analyzed in the ImageJ®.

The construction of the last support base was based on manipulation and all slices were photographed by a digital camera (Digital Camera T3i, Canon®, Japan) coupled to a lens (Lens EF 100 mm f/2.8l Macro IS USM, Canon®, Japan), both supported and stabilized by a tripod (Tripod TG-6660TR, Targus®, U.S.A.), standardizing the capture of adequately identified images. A single operator analyzed these using the ImageJ® processing software (ImageJ® 1.50b, National Institutes of Health, Bethesda, U.S.A.). The points analyzed were: marginal opening (M), gingival-axial angle (G-A), axial region (A), axial-occlusal angle (A-O), and occlusal region (O) (Figure 2A). The operator opened the ImageJ® software and calibrated the program using the image of a photographed ruler with the same standardization used for the sample slices. Next, he measured the predetermined points of each image, recording the values ​​in tables (Figure 2B,D).

### 2.4 Statistical analysis

The values ​​obtained were statistically analyzed using the SPSS 18.0 package (Statistical Package for Social Sciences) and the Excel® software (v. 16.87, Microsoft Office). Descriptive statistics such as mean and standard deviation were considered. To make better use of the data, the Pearson Correlation, ANOVA, and Student's *t*-test statistical tests were used, assuming significance if *p*-value < 0.01.

## 3 Results

The measurement of thicknesses at points M, G-A, A, A-O, and O of each sample obtained using the Replica Technique generated individual values, from which the averages were calculated and statistical analysis of the points between the groups was performed (Table 1).

**Table 1:** Mean thickness (µm) of the points analyzed per group.

	IPS	ZIR	CER	CAD	CAL
M	103.90 ± 28.56^a^	89.6 ± 16.41^a^	104.20 ± 26.68^a^	91.0 ± 61.27^a^	234.10 ± 35.81^b^
G-A	210.8 ± 41.33^a^	218.9 ± 29.82	224.80 ± 73.70	294.40 ± 73.30	312.20 ± 71.64^b^
A	93.2 ± 21.16	124.0 ± 29.55	117.80 ± 50.56	84.50 ± 26.54	79.80 ± 30.28
A-O	247.2 ± 43.67	303.4 ± 43.05^a^	291.60 ± 81.49^a^	308.80 ± 58.13^a^	177.70 ± 91.96^b^
O	282.2 ± 44.68	285.4 ± 44.84	319.50 ± 87.59	329.10 ± 106.38^a^	182.70 ± 135.04^b^

Different letters in the lines indicate a statistically significant difference, *p* < 0.01 (in the same line). IPS, lithium disilicate-reinforced ceramic frameworks; ZIR, ceramic reinforced with zirconia (CAD/CAM); CER, Conventional Lost-Wax Technique; CAD, Vita CAD-Waxx® System; CAL, castable cylinder.

The mean values ​​of marginal openings found in the CAL group (234.1 µm) were the highest among the others, being 161.3% higher than the ZIR group, whose mean values ​​were the lowest (89.6 µm). A parity of 1.6% was observed between the values ​​of the ZIR and CAD groups. The analysis of the G-A angle showed a difference of 48.1% between the groups with the highest and lowest mean values, that is, between the CAL (312.2 µm) and the IPS (210.8 µm), respectively.

In the axial region, the smallest difference (5.89%) was observed between the values ​​of the CAL (79.8 µm) and CAD (84.5 µm) groups; and the greatest disparity between the CAL (79.8 µm) and CER (117.8 µm) groups of 47.82%. Statistically, there was a significant difference (*p* < 0.01) between the values ​​of the CAL group and the other groups, except in the axial region, where the difference between all groups was not significant. The lowest mean value found was at the axial points of the CAL group (79.80 µm), while the highest value occurred in the occlusal region of the CAD group (329.10 µm). The mean for each sample group was calculated and shown in Table 2. In this table, the IPS group exhibited the lowest mean value for internal thickness, i.e., the cement thickness for these infrastructures would be smaller than for the other samples. On the other hand, the largest space for cementation was found in the CAD group.

**Table 2:** Average thickness (µm) of each group of samples.

Groups	IPS	ZIR	CER	CAD	CAL
Average	187.7	204.3	211.6	221.6	197.3

IPS, lithium disilicate-reinforced ceramic frameworks; ZIR, ceramic reinforced with zirconia (CAD/CAM); CER, Conventional Lost-Wax Technique; CAD, Vita CAD-Waxx® System; CAL, castable cylinder.

**Figure 3:**
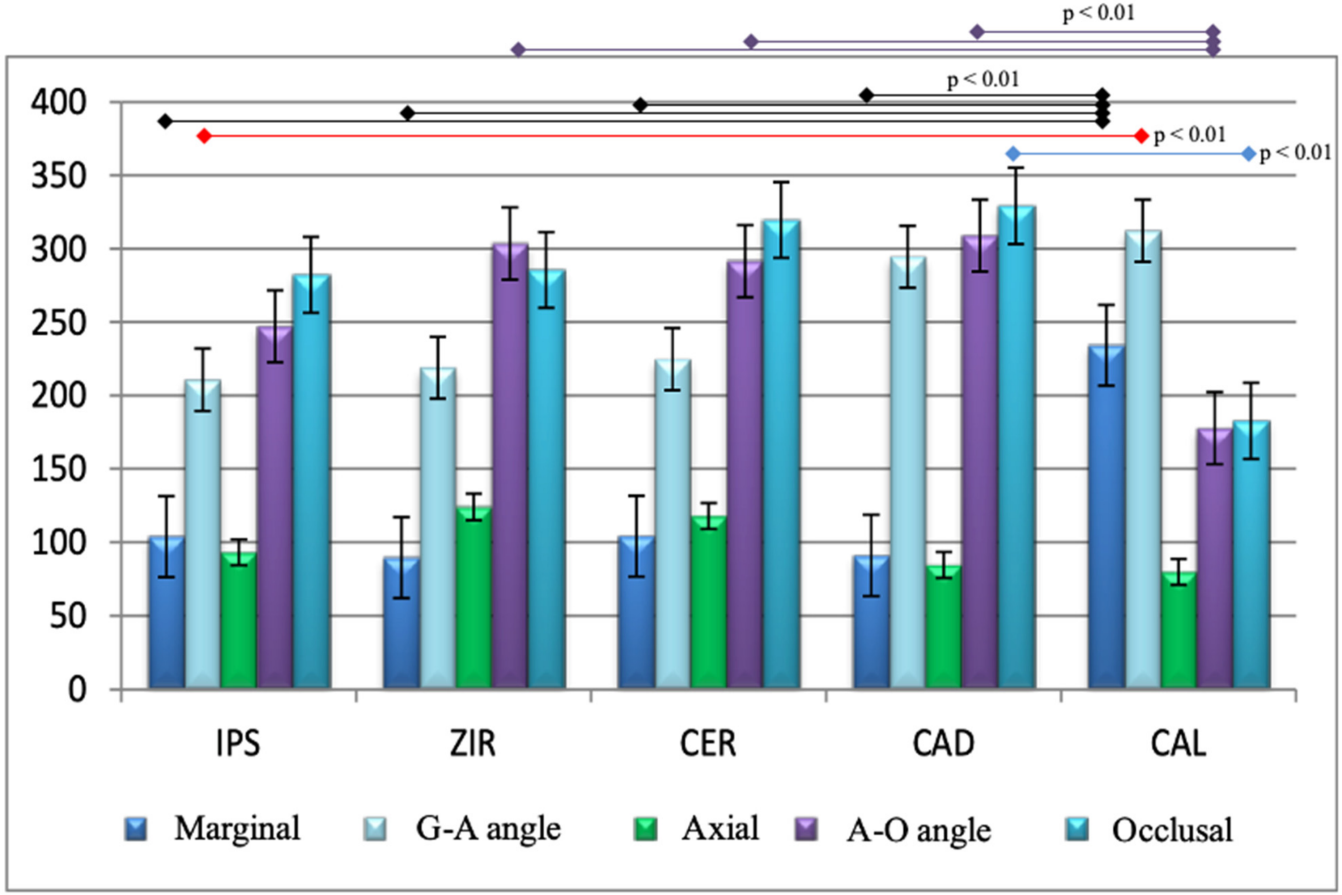
Comparison between the mean values of the points analyzed between the groups. Statistical significance: Purple – A-O angle; Black – Marginal; Red – G-A angle; Blue – Occlusal. IPS, lithium disilicate-reinforced ceramic frameworks; ZIR, ceramic reinforced with zirconia (CAD/CAM); CER, Conventional Lost-Wax Technique; CAD, Vita CAD-Waxx® System; CAL, castable cylinder.

In general, the values ​​obtained at the axial and marginal points of the samples were lower than the other points, especially concerning the angles G-A and A-O (Figure 3).

**Figure 4:**
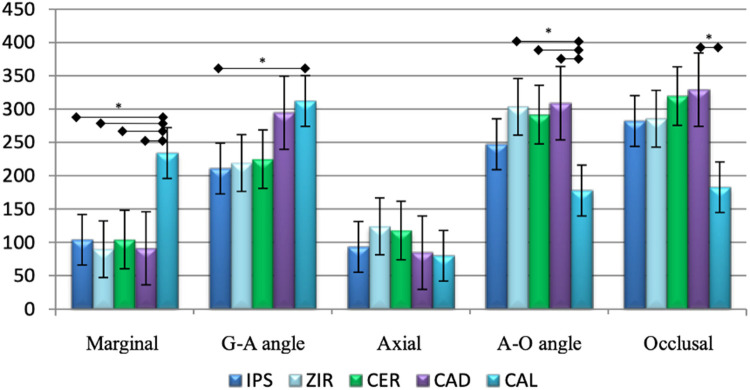
Representation of the averages obtained between the points analyzed. Statistical significance: * = (*p* < 0.01). IPS: lithium disilicate-reinforced ceramic frameworks; ZIR: ceramic reinforced with zirconia (CAD/CAM); CER: Conventional Lost-Wax Technique; CAD: Vita CAD-Waxx® System; CAL: castable cylinder.

According to Figure 4 and Table 1, the analysis of the values ​​obtained in the G-A angles indicates a great disparity between the IPS (210.8 µm) and CAL (312.2 µm) groups, with a difference of 48.1% between the averages. The greatest difference between the groups in the means of the A-O angles was 73.78%, observed between the CAL (177.7 µm) and CAD (308.8 µm) groups; the smallest difference was 39.11%, identified between the CAL (177.7 µm) and IPS (247.2 µm) groups. Likewise, the greatest inequality between the groups of measurements on the occlusal surface was also observed between the means of the CAL (182.7 µm) and CAD (329.1 µm) groups, with a difference of 80.13% between the values.

Figure 2 also shows the reduced values ​​found at the marginal and axial points in all groups analyzed. The overall average found at the marginal points was 124.56 µm, and at the axial points, it was 99.86 µm. The occlusal region exhibited the highest internal space values, with an average of 279.78 µm. When analyzing the groups separately, the resulting values ​​for the IPS group (Table 1) indicate spaces of more than 150 µm at the G-A, A-O angles, and in the O region. The axial and marginal regions evaluated presented adaptation values ​​close to 100 µm. The analyses of the occlusal region and the G-A and A-O angles of the ZIR group (Table 1) showed average internal space values ​​between 218.9 µm and 303.4 µm. In contrast, the marginal and axial regions ranged from 89.6 µm to 124 µm. Regarding the CER group (Table 1), all mean values ​​found were greater than 100 µm, with the occlusal points and angles G-A and A-O being between 224.8 µm and 319.5 µm and the axial and marginal points not exceeding the mean of 118 µm.

The mean occlusal value of the CAD group was the highest shown in Table 1, 329.1 µm, accompanied by one of the largest standard deviations (106.38) found. The A-O and G-A angles also expressed high means of 308.8 µm and 294.4 µm, respectively. Once again, the marginal and axial values ​​did not exceed 100 µm; otherwise, the CAL group presented a different pattern of values ​​from the other groups (Figure 2). The G-A angle and the marginal values ​​exceeded 200 µm; the A-O and occlusal angle values ​​remained close to 180 µm; and the axial points exhibited the lowest value found, 79.8 µm (Table 1). The comparison of intra-group values ​​was highly significant (*p* < 0.01); it means that the values ​​analyzed at each point of the samples presented differences between them.

## 4 Discussion

Numerous studies are available on internal and marginal adaptation of infrastructures on prosthetic abutments (36, 37); therefore, the results are heterogeneous due to the different techniques employed, the variations in sample numbers used, and the types of systems compared in each study (11, 15, 38). According to Hung et al. (14), the most important criterion considered by dentists to evaluate the clinical acceptability of prosthetic restorations is the marginal adaptation. The literature indicates a direct relationship between marginal discrepancies between fixed prostheses and periodontal/peri-implant tissue inflammation (13, 15, 33, 39). Some authors report a considerable increase in the vertical marginal gap between the prosthetic parts and the preparation immediately after cementation; the damage caused by the dissolution of cement of prosthetic restorations on the biological tissues is well established. When part of the material deteriorates, a space is formed below the prosthesis, allowing the infiltration of cariogenic bacteria, whose acidic products lead to the loss of the cementation material and possible displacement of the part, making its replacement essential. As the marginal gap increases (open margin), so does cement degradation (22, 38, 40).

The average adaptation values ​​of infrastructures found in the literature vary considerably. Some authors report satisfactory values ​​of up to 100 µm (16, 17, 41, 42); others consider marginal and internal open margins of up to 120 µm to be acceptable (15, 19, 43); Komine et al. (20) considered the variation from 86 µm to 154 µm to be clinically acceptable, whereas Miura et al. (21) admit the internal variation from 41 µm to 141 µm to be satisfactory. Thus, there is still no consensus in the literature regarding the values ​​of internal/marginal adaptation of the infrastructures. Then, for the internal fit, an internal gap between 50 and 100 µm for indirect restorations cemented by conventional cement and an internal gap of not more than 100 µm (44) to 150 µm (45) for indirect restorations cemented by adhesive cement was recommended, being considered clinically acceptable misfit value.

The ideal space for cementation varies from 25 µm to 40 µm, depending on the cement used. However, these dimensions are not easily obtained in clinical practice. Some values ​​of internal spaces are suggested for each type of technology used; e.g., some authors admit marginal discrepancies of up to 90 µm for parts developed by CAD/CAM systems (11, 21, 38, 42). Although the authors suggested wide variations in internal adaptation, they restricted clinical acceptability to between 100 µm and 150 µm. In other words, this variation is present in most of the reviewed studies, with the minimum value referring to the smallest thickness of cement, considering the cement used; and the maximum value related to the maximum possible open margin without dissolving the cementing agent (11, 13, 17, 22, 40).

In the present study, CAD/CAM technology, which has been largely used (46), was adopted to manufacture ceramic infrastructures for the ZIR group, and the CAD group used scanning derived from this same technology to mill the acrylic pattern. Although the overall average thickness was 204.3 µm for the ZIR group and 221.6 µm for the CAD group, the marginal average values ​​were 89.6 µm and 91 µm, respectively. In other words, analyzing the cementation line facing the oral environment, a region considered critical for the integrity of the cementation of the parts, the values ​​are close to those clinically tolerated by the literature consulted (90 µm).

Boitelle et al. (11), in a systematic review studying CAD/CAM systems adaptation, stated that accuracy was an intrinsic property of each manufacturing system. The authors concluded it was possible to obtain internal spaces with values ​​lower than 80 µm, unlike the results of the present research. Even though the CAD/CAM system was designed to reduce manufacturing defects in prosthetic parts, some irregularities may be caused by scanning, the design traced by the software, milling, and the processed material. It is possible that the resolution of the scanning system, the wax relief of the sharp angles, as well as the silver powder film applied to the prosthetic abutment to inhibit reflection during scanning/reading have caused some changes in the internal discrepancies (47). Since the average obtained in the present study refers to several points analyzed along the cementation film, it is possible that the relief of the sharp angles required for scanning the analog abutment and the 30% compensation made by the operator in the software before milling interfered with the final value of maladaptation of the zirconia parts. The machining of ceramic blocks requires an increase of 20% to 30% in the volume of the parts and subsequent sintering and contraction in the same proportion. This is a fact already known and predetermined by systems such as Cercon® Zirconia, VITA®, and IPS e.max®. Therefore, the contraction of the sintering process and the prior compensation of milling should not be part of the variables in the maladaptation of the infrastructures (48).

Wettstein et al. (33) believe that infrastructures made of zirconia tend to have larger internal spaces than metal-ceramic pieces. The authors assumed that as the axial value increases, the marginal opening becomes larger, which is not consistent with the present study since the zirconia pieces presented an average of 124 µm in the axial region and 89.6 µm in the marginal region. Using the Cercon® system, Miura et al. (21) obtained average marginal results close to those of this study, from 70 µm to 80 µm. However, in occlusal regions, the average ranged from 133 µm to 141 µm, and in axial regions, between 41 µm and 45 µm. The authors guarantee the clinical applicability of the pieces manufactured by this CAD/CAM system, considering marginal openings smaller than 100 µm, in agreement with the values ​​of the ZIR group of the present study (average marginal openings of the ZIR group of 89.6 µm), important to avoid cement dissolution.

It is also possible that the excessive variation found between the points of the ZIR group is due to heating during the sintering of the ceramic Berejuk et al. (49) reported the sintering process of milled (pre-sintered) zirconia by the CAD/CAM system. can cause distortions in the material, resulting in misfits of the infrastructure on the prosthetic abutment. According to Colpani et al. (13), the final characteristics of the piece also depend on the homogeneity of the ceramic block and the precision of the software when performing the virtual delineation according to the desired compensation. Any increase in the temperature of the ceramics results in the expansion and contraction of the material. These variations are compensated for in the CAM stage, which is in the milling of the porcelain, through adjustments in the CAD stage. The fact that this process is conducted by equipment and does not depend on the skills of an operator may explain the lower standard deviation values ​​found within the ZIR group, revealing the greater precision among its samples when compared to the other groups (9, 23).

Due to the recent incorporation of the CAD/Waxx® system in Dentistry, no study was found regarding the evaluation and adaptation of infrastructures manufactured using this technology. However, it is possible to consider some aspects inherent to the CAD/CAM system and the lost wax technique present during this process. Thus, the significant average value of the thickness obtained in the samples of the CAD group can be explained by interferences such as the manipulation of the software by the operator when stipulating 70 µm for the internal space, as well as all the factors related to the expansion/contraction of the coating and also of the metal used.

All metal parts subjected to casting, i.e., the CER, CAD, and CAL groups, were subject to variations in thickness and internal space due to factors such as expansion of the coating material during the inclusion stage, expansion of the metal during casting, subsequent contraction of the metal resulting from its cooling, and the operator's skills in controlling the time, temperature, and quantity of materials used in each phase of the processes. Although metal infrastructures have a greater number of laboratory steps and more variables capable of resulting in changes in the adaptation of the parts, the CAL group presented a lower average thickness value (197.3 µm). This same group presented the lowest values ​​in the occlusal (182.7 µm) and axial-occlusal (177.7 µm) regions but the largest spaces in the marginal line (234.1 µm) among the groups. This last value, in addition to being clinically undesirable for the adaptation of infrastructures, is not in accordance with the literature that shows results of up to 30 µm (38, 50, 51). The occlusal region showed high values ​​in all groups, differing statistically between two of them, CAD with the highest average found (329.10 µm) and CAL with the lowest (182.70 µm). The increased space in the occlusal region can cause harm to the longevity of the prosthesis, such as the reduction in the amount of ceramic to be applied to the infrastructure; the presence of premature contact against the antagonist element; transmission of occlusal force and consequent cracking or fracture of the piece or even favoring shearing forces allowing the loosening of the prosthetic crown (52). Defects during the casting process of metal parts can interfere with their misalignment on implants. Falcão-Filho (53) and Cardoso et al. (51) evaluated the vertical adaptation of the intermediate/prosthetic cylinder interface and did not find values ​​greater than 31.82 µm using castable cylinders. These values ​​were much lower than those found in the present study. Furthermore, the occlusal region of the CAD and CAL groups expressed the highest standard deviations (±106.38 and ±135.04, respectively) in the punctual evaluation between groups, which suggests the need for more specific analyses on the casting process of the metal groups. The interference of the operator's manual skill during the steps of manufacturing the metal structures and the reliefs made in the angles for scanning the prosthetic abutment for the manufacture of the ceramic infrastructures may be determining factors in the increase in the internal thickness of both the A-O and G-A angles, as well as the occlusal region, which expressed equally high values ​​in most groups.

The infrastructures of the CER group, obtained by the conventional lost-wax technique, presented M, A, O, and mean adaptation values ​​higher than those found in the study by Vojdani et al. (47), in which the authors also used this manufacturing process for metal infrastructures. A study (54) reported the difficulty in obtaining perfect marginal adaptation through the lost-wax technique because, in addition to being an indirect prosthetic procedure, it involves the use of different materials, each with distinct physical and chemical properties. According to Wataha (55), these materials undergo dimensional changes, preventing the infrastructures from having the same dimensions as the previously made patterns. Even so, Yamaguti (56) assures that the use of Bella Vest® coating, employed in his work and also in the present study, was capable of resulting in cast parts with adequate cervical adaptation. Wax is another important material used in this procedure. Its alteration characterizes the most critical problem observed during the making or removal of the pattern. These distortions are due to thermal variations, the release of tensions due to cooling, air entrapment, sculpting, removal, and the time of burning and storage (35).

The casting process requires the metal alloy to have some properties, such as biocompatibility and structural properties, that guarantee the adequate function and durability of the restoration. However, the contraction suffered by dental alloys resulting from the change in physical state from liquid to solid can interfere with the marginal opening and the maladaptation of the infrastructures (55, 57).

The IPS group presented the lowest average thickness value among the silicone films (187.7 µm), influenced by the reduced spaces observed at the axial (93.2 µm) and marginal (103.9 µm) points. These values, translated into clinical practice, presuppose the smallest amount of cement used to seat the pieces, as well as the smallest fraction of cement exposed to the oral environment. Some authors (18, 23) found mean marginal values ​​for zirconia frameworks to be lower than for lithium disilicate frameworks. These findings are in line with the present study, comparing both ceramic groups.

The thermo-injection process requires some heating steps, such as firing the acrylic pattern and casting the ceramic tiles for flow within the lining. The expansion and contraction of the material resulting from these steps increase the risk of causing undesirable dimensional changes in the final piece. In the specific evaluation of this research, adaptations with values ​​greater than 250 µm were found at the occlusal points (279.78 µm), A-O angle (265.74 µm), and G-A angle (252.22 µm). These are critical locations that are susceptible to changes during the construction stages of the parts, whether in metals or ceramics, mainly due to angular reliefs, expansion/contraction of the materials, and operator control/skill. Considering that castable cylinders are prefabricated artifacts and only required the lost-wax technique to obtain the final infrastructure, the value of the internal thickness was in accordance with the assumption that the fewer stages there are in a process, the lower the risk of misfits in the final part. However, the IPS group exhibited the lowest value among the groups, despite the process being one of the most extensive. Following this same line of reasoning, the CAD (221.6 µm) and CER (211.6 µm) groups presented higher average misfit values, making up the groups with the largest number of stages in the manufacturing process (38). In general, the marginal and axial points were the only ones that provided values ​​appropriate for clinical application, according to the variation admitted by the literature consulted, except for the marginal openings of the group that used calcinable cylinders. The other internal points presented values ​​greater than 150 µm, which goes beyond the principles that govern the integrity and longevity of the infrastructure. Since the marginal space is the greatest concern of the dentist regarding the internal adaptation of prosthetic parts, all groups provided satisfactory conditions for clinical use, except for the CAL group.

The clinical application of the replica technique can be suggested in cases in which the dentist needs to observe more precisely the configuration of the film resulting from replicating the entire cementation layer or even a dental preparation. Similarly, Hollweg et al. (29) and Machado et al. (30) describe the use of this printing technique to check for interference areas that prevent the prosthetic part from fitting onto the preparation, using fluid addition silicone (26, 32). In this study, the analog abutment was used to make the infrastructures and the components to evaluate the internal space of the samples. This is because the closer to clinical reality, the more reliable the scientific work becomes. However, the difference between the materials used to manufacture both prosthetic abutments must be considered since the analog abutment was made of brass and the component of titanium, suggesting over- or underestimation of the adaptation values ​​of the infrastructures on the component. There is a need for scientific studies explaining such differences, as well as the limitations of using both abutments in *in vitro* studies, in order to minimize or justify the results of exacerbated maladaptations. It is important to consider that, as this is an *in vitro* study, the values ​​obtained and compared with the literature may present some disagreement with those observed in clinical practice since the infrastructures were made from a standardized prosthetic pillar and not inserted into the oral environment.

### 4.1 Limitations, strengths, and future research

This study had some strengths and limitations: it compared measurements of 5 different types of frameworks relevant to the clinical routine; otherwise, it used only one technique (the Replica Technique) for assessment, and the number of materials used (50 frameworks divided into five groups) can be considered low. For future research, it is recommended that the sample size be increased and other techniques, such as digital techniques, be used to compare with Replica. Using a scanner and designing software avoids multiple replications and transfers of data from one software to another (when using software of the same company), which could cause errors resulting in decreased accuracy (58).

Based on the methodology used, the general average obtained for the thicknesses of each group (IPS: 187.7 µm; ZIR: 204.3 µm; CER: 211.6 µm; CAD: 221.6 µm; CAL: 197.3 µm) permitted to conclude that the average adaptation value, closest to the clinically acceptable variation in the literature, was obtained in IPS. The axial region was the only evaluated site that demonstrated similar adaptation values ​​between the infrastructure groups. The values ​​observed in the occlusal region in all groups were higher than those found in the literature, constituting an aspect of great concern in the fitting of the prosthetic piece. The angles evaluated (A-O and G-A) resulted in high internal spaces, suggesting a possible association with process steps, whether they were relief of sharp angles or operator interference in the preparation of patterns prior to casting. Although several authors suggest reasons that may lead to undesirable maladaptation of the pieces, such as the number of process steps, heating of the ceramic material, relief of sharp angles before scanning, or the need for manual intervention by an operator, further studies are needed to deepen the search and eliminate the suggested hypotheses.

## Data Availability

The raw data supporting the conclusions of this article will be made available by the authors, without undue reservation.
